# Epidemiology of cardiogenic shock using the Shock Academic Research Consortium (SHARC) consensus definitions

**DOI:** 10.1093/ehjacc/zuae098

**Published:** 2024-08-29

**Authors:** David D Berg, Erin A Bohula, Siddharth M Patel, Carlos E Alfonso, Carlos L Alviar, Vivian M Baird-Zars, Christopher F Barnett, Gregory W Barsness, Courtney E Bennett, Sunit-Preet Chaudhry, Christopher B Fordyce, Shahab Ghafghazi, Umesh K Gidwani, Michael J Goldfarb, Jason N Katz, Venu Menon, P Elliott Miller, L Kristin Newby, Alexander I Papolos, Jeong-Gun Park, Matthew J Pierce, Alastair G Proudfoot, Shashank S Sinha, Lakshmi Sridharan, Andrea D Thompson, Sean van Diepen, David A Morrow

**Affiliations:** Levine Cardiac Intensive Care Unit, Cardiovascular Division, Department of Medicine, Brigham and Women’s Hospital, Harvard Medical School, 60 Fenwood Road, Suite 7022, Boston, MA 02115, USA; Levine Cardiac Intensive Care Unit, Cardiovascular Division, Department of Medicine, Brigham and Women’s Hospital, Harvard Medical School, 60 Fenwood Road, Suite 7022, Boston, MA 02115, USA; Levine Cardiac Intensive Care Unit, Cardiovascular Division, Department of Medicine, Brigham and Women’s Hospital, Harvard Medical School, 60 Fenwood Road, Suite 7022, Boston, MA 02115, USA; Division of Cardiovascular Medicine, University of Miami Hospital, University of Miami Miller School of Medicine, Miami, FL, USA; Leon H Charney Division of Cardiology, New York University Grossman School of Medicine, New York, NY, USA; Levine Cardiac Intensive Care Unit, Cardiovascular Division, Department of Medicine, Brigham and Women’s Hospital, Harvard Medical School, 60 Fenwood Road, Suite 7022, Boston, MA 02115, USA; Division of Cardiology, Department of Medicine, University of California San Francisco, San Francisco, CA, USA; Department of Cardiovascular Medicine, Mayo Clinic, Rochester, MN, USA; Lehigh Valley Heart Institute, Department of Cardiology, Allentown, PA, USA; Department of Medicine, St Vincent Heart Center, Indianapolis, IN, USA; Division of Cardiology, Department of Medicine, Vancouver General Hospital and The Centre for Cardiovascular Innovation, University of British Columbia, Vancouver, BC, Canada; Division of Cardiovascular Medicine, Department of Medicine, University of Louisville School of Medicine, Louisville, KY, USA; Division of Cardiology, Zena and Michael A. Wiener Cardiovascular Institute, Icahn School of Medicine at Mount Sinai, New York, NY, USA; Division of Cardiology, Jewish General Hospital, McGill University, Montreal, QC, Canada; Leon H Charney Division of Cardiology, New York University Grossman School of Medicine, New York, NY, USA; Department of Cardiovascular Medicine, Cleveland Clinic Foundation, Heart and Vascular Institute, Cleveland, OH, USA; Section of Cardiovascular Medicine, Yale University, New Haven, CT, USA; Division of Cardiology, Department of Medicine and Duke Clinical Research Institute, Duke University, Durham, NC, USA; Departments of Cardiology and Critical Care, MedStar Washington Hospital Center, Washington, DC, USA; Levine Cardiac Intensive Care Unit, Cardiovascular Division, Department of Medicine, Brigham and Women’s Hospital, Harvard Medical School, 60 Fenwood Road, Suite 7022, Boston, MA 02115, USA; Northwell Cardiovascular Institute, New Hyde Park, NY, USA; Department of Perioperative Medicine, Barts Heart Centre, Barts Health NHS Trust, London, UK; Inova Schar Heart and Vascular, Inova Fairfax Medical Campus, Falls Church, VA, USA; Division of Cardiovascular Medicine, Department of Medicine, Emory University School of Medicine, Atlanta, GA, USA; Division of Cardiovascular Medicine, Department of Medicine, University of Michigan, Ann Arbor, MI, USA; Department of Critical Care Medicine and Division of Cardiology, Department of Medicine, University of Alberta, Edmonton, AB, Canada; Levine Cardiac Intensive Care Unit, Cardiovascular Division, Department of Medicine, Brigham and Women’s Hospital, Harvard Medical School, 60 Fenwood Road, Suite 7022, Boston, MA 02115, USA

**Keywords:** Cardiogenic shock, Epidemiology, Cardiac intensive care unit

## Abstract

**Aims:**

The Shock Academic Research Consortium (SHARC) recently proposed pragmatic consensus definitions to standardize classification of cardiogenic shock (CS) in registries and clinical trials. We aimed to describe contemporary CS epidemiology using the SHARC definitions in a cardiac intensive care unit (CICU) population.

**Methods and results:**

The Critical Care Cardiology Trials Network (CCCTN) is a multinational research network of advanced CICUs coordinated by the TIMI Study Group (Boston, MA). Cardiogenic shock was defined as a cardiac disorder resulting in SBP < 90 mmHg for ≥30 min [or the need for vasopressors, inotropes, or mechanical circulatory support (MCS) to maintain SBP ≥ 90 mmHg] with evidence of hypoperfusion. Primary aetiologic categories included acute myocardial infarction-related CS (AMI-CS), heart failure-related CS (HF-CS), and non-myocardial (secondary) CS. Post-cardiotomy CS was not included. Heart failure-related CS was further subcategorized as *de novo* vs. acute-on-chronic HF-CS. Patients with both cardiogenic and non-cardiogenic components of shock were classified separately as mixed CS. Of 8974 patients meeting shock criteria (2017–23), 65% had isolated CS and 17% had mixed shock. Among patients with CS (*n* = 5869), 27% had AMI-CS (65% STEMI), 59% HF-CS (72% acute-on-chronic, 28% *de novo*), and 14% secondary CS. Patients with AMI-CS and *de novo* HF-CS were most likely to have had concomitant cardiac arrest (*P* < 0.001). Patients with AMI-CS and mixed CS were most likely to present in more severe shock stages (SCAI D or E; *P* < 0.001). Temporary MCS use was highest in AMI-CS (59%). In-hospital mortality was highest in mixed CS (48%), followed by AMI-CS (41%), similar in *de novo* HF-CS (31%) and secondary CS (31%), and lowest in acute-on-chronic HF-CS (25%; *P* < 0.001).

**Conclusion:**

SHARC consensus definitions for CS classification can be pragmatically applied in contemporary registries and reveal discrete subpopulations of CS with distinct phenotypes and outcomes that may be relevant to clinical practice and future research.

**Key points**

- In this large observational cohort of cardiogenic shock (CS) admissions from the CCCTN Registry, SHARC definitions for CS classification were pragmatically applied, revealing discrete subpopulations of CS with distinct phenotypes and outcomes.
- In-hospital mortality was highest in mixed CS (48%), followed by acute myocardial infarction-related CS (AMI-CS; 41%), similar in *de novo* heart failure-related CS (HF-CS; 31%) and secondary CS (31%), and lowest in acute-on-chronic HF-CS (25%).
- These foundational epidemiologic data are relevant to clinical practice and provide benchmarks for future clinical research and quality improvement efforts.

## Introduction

Cardiogenic shock (CS) is a heterogeneous syndrome with important variation in aetiology,^1^ chronicity,^2^ end-organ injury,^3^ haemodynamic profile,^4,5^ and severity.^6,7^ One hypothesized explanation for the challenges with establishing a benefit of many studied interventions in CS is that trials have inadequately accounted for this heterogeneity.^8^ To this end, there have been efforts to standardize grading of CS severity [e.g. Society for Cardiovascular Angiography and Intervention (SCAI) shock stage] and to develop a common taxonomy for describing CS subtypes and outcomes.

The Shock Academic Research Consortium (SHARC) is a multi-stakeholder group that developed consensus definitions for classifying CS populations in clinical trials and registries.^9^ The SHARC framework has not yet been applied in a contemporary registry. The aim of this study was to describe contemporary CS epidemiology using the SHARC classifications.

## Methods

### Data collection

The Critical Care Cardiology Trials Network (CCCTN) is a multinational network of cardiac intensive care units (CICUs) coordinated by the TIMI Study Group (Boston, MA). The CCCTN Registry methods have been reported.^10^ From 2017–23, participating centres contributed consecutive medical CICU admissions during annual 2-month collection periods. The CCCTN Registry protocol and waiver of informed consent were approved by the IRB at each centre.

### Shock classification

Patients were classified according to their initial presenting shock state. Consistent with the SHARC definition, CS was defined clinically in the CCCTN Registry as a cardiac disorder that results in haemodynamic impairment [i.e. SBP < 90 mmHg for ≥30 min or the need for vasopressors, inotropes, or mechanical circulatory support (MCS) to maintain SBP ≥ 90 mmHg] with evidence of hypoperfusion (see Supplemental Methods). Primary aetiologic categories included acute myocardial infarction-related CS (AMI-CS), heart failure-related CS (HF-CS), and non-myocardial (secondary) CS (e.g. cardiac tamponade and severe valvular disease). Heart failure-related CS was subcategorized as *de novo* (i.e. related to acute myocardial dysfunction known or suspected to be new) vs. acute-on-chronic (i.e. decompensation of chronic HF). Mixed CS classification is described in the Supplemental Methods.

### Data analysis

Standardized data elements (e.g. demographics, medical history, and presenting clinical characteristics), shock-specific variables [e.g. whether CS occurred in the setting of cardiac arrest (SHARC modifier) and SCAI stage], and in-hospital mortality were summarized according to profile and aetiology. Results are reported as counts and percentages for categorical variables and medians (25th–75th percentiles) for continuous variables. Absolute 95% confidence intervals (CI) were calculated using the binomial method.

## Results

### Shock classification

**Figure 1:**
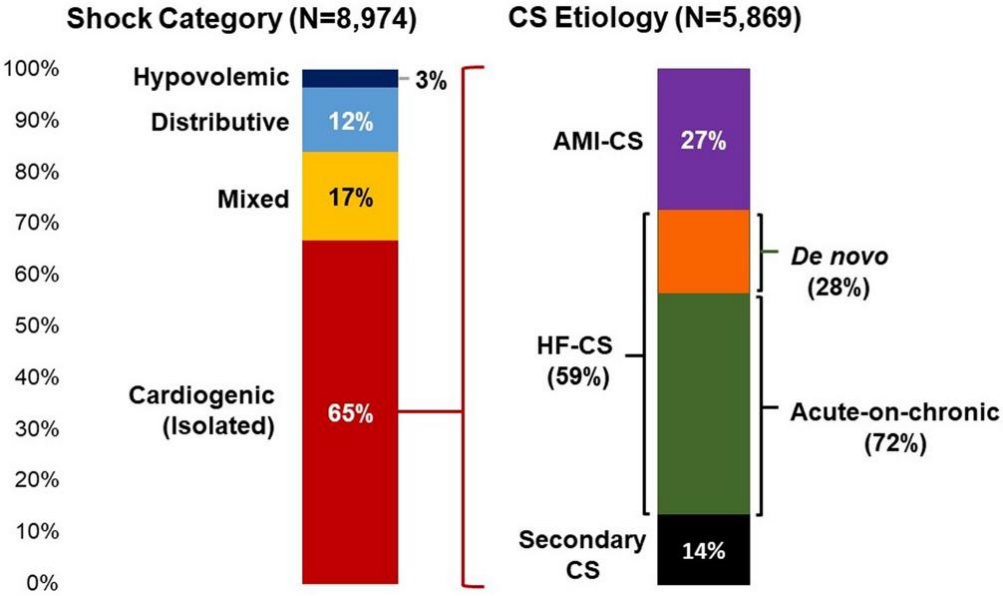
Shock profile and aetiology in a cardiac intensive care unit (CICU) population. Shock cases were classified according to primary haemodynamic profile. Cardiogenic shock (CS) cases were sub-classified according to primary aetiology. Mixed shock indicates that more than one shock category substantially contributed to the haemodynamic profile, and mixed CS refers to mixed shock cases with a known cardiogenic component (∼93% of all mixed shock). Cases for which the shock category was uncertain (*n* = 188; 2% of all shock cases) are not included in the figure. AMI-CS, acute myocardial infarction-related cardiogenic shock; CS, cardiogenic shock; HF-CS, heart failure-related cardiogenic shock.

Among the 8974 patients meeting shock criteria, 65% had CS and 17% had mixed shock (*Figure 1*). Among patients with CS (*n* = 5869), 27% had AMI-CS (65% STEMI), 59% had HF-CS (72% acute-on-chronic, 28% *de novo*), and 14% had secondary CS. Leading causes of secondary CS (*n* = 799) included arrhythmia (33%), severe valvular disease (27%), pericardial disease (9%), and pulmonary vascular disease (8%). Among patients with confirmed mixed CS (*n* = 947; 2019–23), comprising 93% of all mixed shock, 19% of cases were related to AMI (53% STEMI). Five per cent of isolated CS cases were known to have evolved to mixed CS, and 3% of mixed CS cases to isolated CS.

### Clinical characteristics and cardiac intensive care unit resource utilization by shock aetiology

Clinical characteristics and CICU resource utilization are summarized by shock aetiology in *Table 1*. Patients with AMI-CS and *de novo* HF-CS were most likely to have had CS in the setting of cardiac arrest (33% and 31%, respectively).

**Table 1:** Characteristics and cardiac intensive care unit resource utilization by shock aetiology

	AMI-CS (*N* = 1597)	*De novo* HF-CS (*N* = 968)	Acute-on-chronic HF-CS (*N* = 2504)	Secondary CS (*N* = 799)	Mixed CS (*N* = 947^a^)
Demographics					
Age, median (25th–75th), y	68 (59–77)	62 (50–73)	63 (54–72)	69 (58–78)	69 (59–77)
Female sex, *n* (%)	492 (30.8%)	367 (37.9%)	714 (28.5%)	370 (46.3%)	344 (36.3%)
Race					
White, *n* (%)	928 (58.1%)	571 (59.0%)	1412 (56.4%)	501 (62.7%)	553 (58.4%)
Black, *n* (%)	166 (10.4%)	190 (19.6%)	668 (26.7%)	115 (14.4%)	169 (17.8%)
Other, *n* (%)	503 (31.5%)	207 (21.4%)	424 (16.9%)	183 (22.9%)	225 (23.8%)
BMI, median (25th–75th), kg/m^2^	27.8 (24.4–31.8)	26.8 (23.2–31.6)	27.3 (23.4–32.2)	27.7 (23.9–33.0)	27.3 (23.5–32.6)
Comorbidities					
Diabetes mellitus, *n* (%)	719 (45.0%)	266 (27.5%)	966 (38.6%)	256 (32.0%)	357 (37.7%)
Coronary artery disease, *n* (%)	604 (37.8%)	245 (25.3%)	1070 (42.7%)	237 (29.7%)	337 (35.6%)
Heart failure, *n* (%)	315 (19.7%)	0 (0%)	2504 (100.0%)	344 (43.1%)	506 (53.4%)
Severe valvular disease, *n* (%)	66 (4.1%)	81 (8.4%)	499 (19.9%)	224 (28.0%)	190 (20.1%)
Pulmonary hypertension, *n* (%)	18 (1.1%)	54 (5.6%)	275 (11.0%)	98 (12.3%)	103 (10.9%)
Chronic kidney disease, *n* (%)	316 (19.8%)	165 (17.0%)	996 (39.8%)	214 (26.8%)	318 (33.6%)
On dialysis, *n* (%)	70 (22.2%)	33 (20.1%)	101 (10.2%)	48 (22.4%)	81 (25.5%)
Presenting clinical features					
SOFA score, median (25th–75th)	8 (5–10)	7 (5–11)	6 (4–9)	7 (5–10)	9 (7–12)
SOFA score ≥ 8, *n* (%)	804 (50.3%)	474 (49.0%)	938 (37.5%)	369 (46.2%)	634 (66.9%)
SCAI stage					
B, *n* (%)	126 (10.8%)	96 (13.2%)	233 (12.8%)	94 (16.0%)	56 (6.0%)
C, *n* (%)	557 (47.6%)	395 (54.1%)	1143 (62.9%)	330 (56.2%)	484 (51.5%)
D, *n* (%)	349 (29.9%)	173 (23.7%)	382 (21.0%)	105 (17.9%)	300 (31.9%)
E, *n* (%)	137 (11.7%)	66 (9.0%)	59 (3.2%)	58 (9.9%)	100 (10.6%)
Presenting LVEF < 30%, *n* (%)	777 (48.7%)	591 (61.1%)	1934 (77.3%)	151 (18.9%)	391 (41.3%)
Pattern of ventricular involvement					
Left ventricular	1092 (68.4%)	521 (53.8%)	1379 (55.1%)	—	240 (47.8%)
Right ventricular	74 (4.6%)	98 (10.1%)	133 (5.3%)	—	45 (9.0%)
Biventricular	298 (18.7%)	349 (36.1%)	992 (39.6%)	—	125 (24.9%)
Non-ventricular	132 (8.3%)	0 (0.0%)	0 (0.0%)	—	92 (18.3%)
Preceding cardiac arrest, *n* (%)	530 (33.2%)	296 (30.6%)	271 (10.8%)	187 (23.4%)	239 (25.2%)
Laboratory values (admission)					
Lactate, median (25th–75th), mmol/L	2.9 (1.7–5.6)	3.0 (1.7–6.0)	2.2 (1.3–3.8)	2.7 (1.6–4.9)	2.9 (1.7–5.7)
Lactate ≥ 4 mmol/L, *n* (%)	536 (37.5%)	327 (38.1%)	513 (23.9%)	234 (33.6%)	336 (38.3%)
eGFR, median (25th–75th), mg/dL	54 (34–74)	51 (31–72)	42 (26–61)	44 (26–63)	39 (21–62)
eGFR < 60, *n* (%)	899 (59.0%)	585 (62.6%)	1803 (73.8%)	557 (71.8%)	660 (73.3%)
ICU resource utilization					
CICU LOS, median (IQR), days	4.3 (1.9–8.3)	4.7 (2.1–8.2)	5.6 (2.8–11.0)	3.4 (1.7–6.7)	4.8 (2.2–10.2)
Mechanical ventilation, *n* (%)	1045 (65.4%)	494 (51.0%)	783 (31.3%)	369 (46.2%)	631 (66.6%)
Acute RRT, *n* (%)	244 (15.3%)	136 (14.0%)	330 (13.2%)	89 (11.1%)	230 (24.3%)
Pulmonary artery catheter, *n* (%)	570 (35.7%)	349 (36.1%)	1145 (45.7%)	138 (17.3%)	291 (30.7%)
Shock management					
VIS at 4 h	5.0 (0.0–19.8)	4.0 (0.0–13.5)	3.8 (2.0–7.5)	2.5 (0.0–10.0)	10.8 (4.0–25.9)
VIS at 24 h	3.0 (0.0–14.0)	3.0 (0.0–9.0)	3.8 (1.3–7.3)	0.0 (0.0–6.5)	8.0 (2.0–20.0)
MCS, *n* (%)	934 (58.5%)	316 (32.6%)	842 (33.6%)	110 (13.8%)	188 (19.9%)
IABP, *n* (%)	648 (69.4%)	191 (60.4%)	547 (65.0%)	66 (60.0%)	108 (57.4%)
Impella, *n* (%)	338 (36.2%)	94 (29.7%)	216 (25.7%)	20 (18.2%)	60 (31.9%)
TandemHeart, *n* (%)	8 (0.9%)	8 (2.5%)	50 (5.9%)	5 (4.5%)	6 (3.2%)
VA-ECMO, *n* (%)	119 (12.7%)	73 (23.1%)	82 (9.7%)	29 (26.4%)	20 (10.6%)
Surgical (non-durable) VAD, *n* (%)	2 (0.2%)	4 (1.3%)	11 (1.3%)	4 (3.6%)	2 (1.1%)

^a^Includes mixed shock cases with a known cardiogenic component; restricted to annual cycles with these details on mixed shock (2019–23). AMI-CS, acute myocardial infarction-related cardiogenic shock; BMI, body mass index; CICU, cardiac intensive care unit; CS, cardiogenic shock; eGFR, estimated glomerular filtration rate; HF-CS, heart failure-related cardiogenic shock; IABP, intra-aortic balloon pump; LOS, length of stay; LVEF, left ventricular ejection fraction; MCS, mechanical circulatory support; RRT, renal replacement therapy; SCAI, Society for Cardiovascular Angiography and Intervention; SOFA, Sequential Organ Failure Assessment; VA-ECMO, veno-arterial extracorporeal membrane oxygenation; VAD, ventricular assist device; VIS, vasoactive-inotropic score.

The majority of isolated and mixed CS was SCAI stage C (56% and 51%, respectively) with variation by aetiology (*P* < 0.001; *Table 1* and Supplementary material online, *Figure S1*). Among CS subtypes, patients with AMI-CS and mixed CS were most likely to present in SCAI stage D or E. Patients with AMI-CS and mixed CS also had the highest SOFA scores and vasoactive medication requirements (*Table 1*). Biventricular failure was most common in acute-on-chronic HF-CS, and this group tended to have the lowest presenting LVEF.

The use of advanced CICU therapies was common (*Table 1*). Temporary MCS use was highest in AMI-CS (59%). Rates of mechanical ventilation were highest in AMI-CS and mixed CS (65% and 67%, respectively). Rates of acute renal replacement therapy were similar across isolated CS categories (11–15%), but higher in patients with mixed CS (24%).

In sensitivity analyses pooling isolated and mixed CS, the distribution of CS aetiologies was unchanged (see Supplementary material online, *Figure S2* and Supplementary material online, *Table S1*). *Mixed shock syndrome* (as defined in SHARC) was most common in *de novo* HF-CS (see Supplementary material online, *Table S2*).

### Mortality by shock aetiology

**Figure 2:**
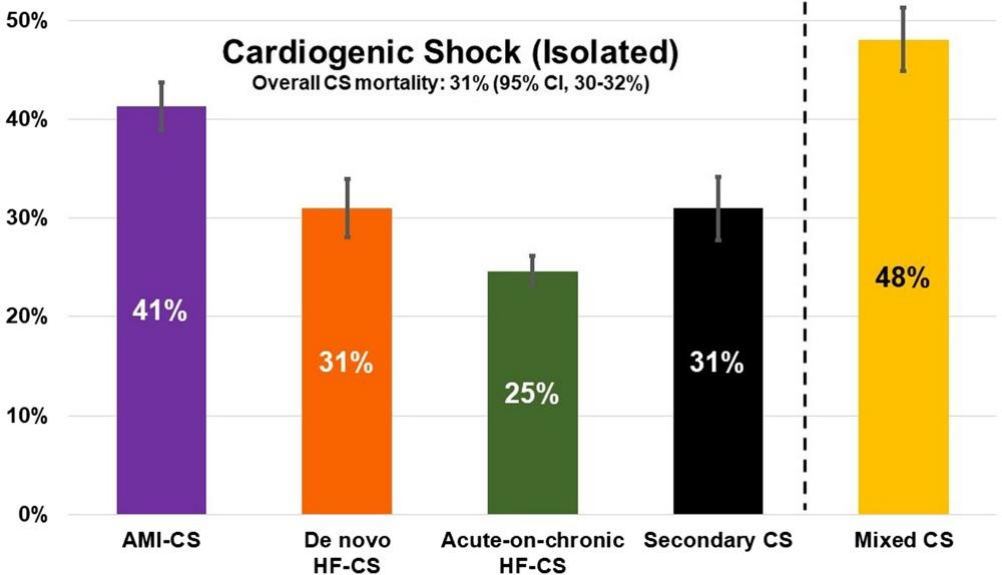
In-hospital mortality by shock profile and aetiology. Error bars indicate the 95% confidence intervals around the in-hospital mortality estimates for each shock category. AMI-CS, acute myocardial infarction-related cardiogenic shock; CS, cardiogenic shock; HF-CS, heart failure-related cardiogenic shock.

In-hospital mortality was higher in patients with mixed CS (48%; 95% CI, 45–51%) as compared to those with isolated CS (*P* < 0.001). Among patients with isolated CS, mortality was highest in patients with AMI-CS (41%; 95% CI, 39–44%), similar in patients with *de novo* HF-CS (31%; 95% CI, 28–34%) and secondary CS (31%; 95% CI, 28–34%), and lowest in patients with acute-on-chronic HF-CS (25%; 95% CI, 23–26%; *Figure 2*). SCAI staging identified stepwise gradients of in-hospital mortality rates across each SHARC CS category (see Supplementary material online, *Figure S3*).

## Discussion

In this analysis from the CCCTN Registry, we applied the SHARC definitions for CS classification, depicting the contemporary landscape of CS and the utility of the SHARC framework for identifying CS subtypes with distinct clinical profiles and outcomes. Building on our previous work^1,2^ but with an updated much larger sample size of shock patients (*n* = 8974), we found that HF-CS is the most common category of CS in contemporary CICUs, and that HF disease chronicity (acute-on-chronic vs. *de novo* HF-CS) is associated with distinct patterns of severity, end-organ injury, and outcomes. In addition, we showed that presenting SCAI shock stage varies across CS categories, with AMI-CS and mixed CS patients presenting with more advanced SCAI shock stages than HF-CS and secondary CS patients. Owing to the large size of our dataset, we were able to estimate in-hospital mortality rates for CS categories more precisely than has been done in previous studies.^1,11^ We found that patients with mixed CS had the highest in-hospital mortality, followed by those with AMI-CS, who in turn had significantly higher mortality than those with HF-CS or secondary CS.

The SHARC definitions thus provide a standardized framework for classifying clinically important CS phenotypes with distinct clinical features and prognoses. These foundational epidemiologic data are not only relevant to clinical practice (e.g. when counselling patients and families) but also provide benchmarks that may be pertinent to quality improvement efforts and to future clinical trial design and interpretation. For instance, the differing results of recently completed trials of temporary MCS highlight the potential relevance of cardiogenic shock phenotype selection in testing specific therapies.^12,13^

Limitations of these analyses should be acknowledged. First, since CCCTN is a registry of medical CICU admissions, we could not include post-cardiotomy CS. Moreover, they reflect the epidemiology of CS in medical CICUs, which may be different from other practice settings. Second, since full details on mixed shock presentations were not collected in early annual cycles, descriptions of our mixed CS population were restricted to 2019–23. Finally, clinical assessment of systemic hypoperfusion can be challenging, particularly in the setting of a missing or normal serum lactate; although it is possible that some patients without shock were included, shock assessments were performed by trained investigators applying criteria aligned with the SHARC definitions.

## Conclusions

SHARC consensus definitions for CS classification can be pragmatically applied in contemporary registries and reveal discrete subpopulations of CS with distinct phenotypes and outcomes.

## Supplementary Material

zuae098_Supplementary_Data

## Data Availability

We encourage parties interested in collaboration and data sharing to contact the corresponding author.
