# Supplementary material for: Epidemiology of cardiogenic shock using the Shock Academic Research Consortium (SHARC) consensus definitions
Source: Eur Heart J Acute Cardiovasc Care. 2024 Aug 29;13(10):709–14. doi: 10.1093/ehjacc/zuae098 (PMC11518926; doi:10.1093/ehjacc/zuae098)
Supplement: zuae098_Supplementary_Data [file zuae098_supplementary_data.docx]

**ONLINE SUPPLEMENT**

**Epidemiology of Cardiogenic Shock in Cardiac Intensive Care Units Using the Framework of the Shock Academic Research Consortium (SHARC) Consensus Definitions: Update from the Critical Care Cardiology Trials Network (CCCTN)**

**Address for Correspondence:**

David D. Berg, MD, MPH

Address: TIMI Study Group

Brigham and Women’s Hospital

60 Fenwood Road, Suite 7022

Boston, MA 02115

Telephone : 617-278-0145

Fax : 617-734-7329

Email : [dberg1@bwh.harvard.edu](mailto:dberg1@bwh.harvard.edu)

Table on Contents

[Supplemental Methods 3](#_Toc164805643)

[Supplemental Table S1 6](#_Toc164805644)

[Supplemental Table S2 7](#_Toc164805645)

[Supplemental Figure S1 8](#_Toc164805646)

[Supplemental Figure S2 9](#_Toc164805647)

[Supplemental Figure S3 10](#_Toc164805648)

# **Supplemental Methods**

*Proposed SHARC definitions of cardiogenic shock*

| **Definition for clinical practice** | Cardiac disorder that results in both clinical and biochemical evidence of sustained tissue hypoperfusion. |
| --- | --- |
| **Definition for clinical trials** | Cardiac disorder that results in a systolic blood pressure <90 mmHg for ≥30 min (or the need for vasopressors, inotropes, or mechanical circulatory support to maintain systolic blood pressure ≥90 mmHg with evidence of hypoperfusion. |

*Summary of SHARC cardiogenic shock classifications*

| **Acute myocardial infarction-related cardiogenic shock (AMI-CS)** | Includes patients with the onset of CS in the setting of acute myocardial infarction (either STEMI or NSTEMI) in which acute ischemia is the primary driver of CS |
| --- | --- |
| **Heart failure-related cardiogenic shock (HF-CS)** | Includes patients with CS related to primary myocardial dysfunction in the absence of acute MI. May be related to left, right, or biventricular dysfunction, and may be ischemic or nonischemic in etiology |
| **De novo** | Related to acute myocardial dysfunction that is known or suspected to be new in onset |
| **Acute-on-chronic** | Acute decompensation of chronic heart failure |
| **Non-myocardial (secondary) CS** | Includes patients with CS related to a primary non-myocardial cardiac cause (e.g., severe valvular disease, pericardial disease, pulmonary embolism, ongoing refractory arrhythmia) |
| **Post-cardiotomy CS** | Includes patients who develop CS in the perioperative period with low cardiac output syndrome as the primary driver of shock |

*CCCTN Shock Definitions*

The assessment of shock was made by trained study staff and the site investigators based on the

entirety of the medical record, including invasive hemodynamic data (when available as part of routine care) and the managing clinician’s diagnoses. Shock and its classification were not adjudicated centrally and thus the local clinical diagnosis took priority. Guidance for determination of shock followed established clinical criteria:

1. Systolic blood pressure (SBP) <90 mmHg for ≥ 30 min (or the need for vasopressors or inotropes to maintain SBP ≥ 90 mmHg) **and**
2. Evidence of end-organ hypoperfusion (ascribed to the hypotension) manifested by any of the following:
3. Altered mental status

ii. Acute kidney injury or oliguria (e.g., urine output <0.5 mL/kg/hour)

iii. Acute hepatic injury (e.g., alanine aminotransferase [ALT] or aspartate aminotransferase [AST] >3x upper limit of normal)

iv. Elevated serum lactate [>2 mmol/L])

v. Cool or mottled extremities

When pulmonary artery catheter data were available, assessment as cardiogenic shock was based on a low cardiac index (i.e., <2.2 mL/min/m2) and elevated left or right ventricular filling pressures (i.e., pulmonary capillary wedge pressure or central venous pressure >15 mmHg). When pulmonary artery catheter data were not available, assessment of cardiogenic shock was based on other supportive clinical features (e.g., pulmonary congestion, elevated jugular venous pressure, low central venous oxygen saturation, echocardiographic evidence of a structural basis for impaired cardiac output, peripheral hypoperfusion on physical exam).

Assessment as distributive shock was based on demonstration of low systemic vascular resistance (e.g., <900 dynes per second/cm5) in the setting of normal or high cardiac output and normal or low ventricular filling pressures. When invasive hemodynamic data were not available, assessment of distributive shock was based on having other supportive clinical features (e.g., low jugular venous pressure, high central venous oxygen saturation, consistent clinical syndrome such as sepsis).

Assessment as hypovolemic shock was based on the presence of reduced preload (e.g., dry mucous membranes, collapsible inferior vena cava on imaging, low ventricular filling pressures on invasive assessment).

Assessment as mixed shock was based on having a hemodynamic profile characterized by low cardiac index (i.e., <2.2 mL/min/m2) and elevated or normal ventricular filling pressures in conjunction with an inappropriately low systemic vascular resistance (e.g., <900 dynes per second/cm5) or, in the absence of invasive hemodynamic data, clinical findings that included the above features of more than one category (e.g. cardiogenic and distributive).

*Classification of Mixed Shock in this Analysis*

In the CCCTN Registry, investigator-classified mixed shock indicates that >1 shock category (e.g., cardiogenic and distributive) substantially contributed to the clinical profile. For this analysis, descriptions of mixed shock were focused on cases with a known cardiogenic component (i.e., “mixed CS”) and thus are restricted to annual cycles with these details on mixed shock (2019-2023). In our primary analyses, mixed CS is reported separately from “isolated” CS (i.e., CS only). Because *mixed shock syndrome* is considered a CS modifier in the SHARC framework, sensitivity analyses were performed describing all CS (including mixed CS cases).

*Equations for derived hemodynamic parameters*

Cardiac power output (CPO) = (MAP*CO)/451

Mean artery pressure (MAP) = (SBP/3) + (2*DBP/3)

Mean pulmonary artery pressure (mPAP) = (PASP/3) + (2*PADP/3)

Pulmonary artery pulsatility index (PAPi) = (PASP-PADP)/RAP

Pulmonary vascular resistance (PVR) = (mPAP-PCWP)/CO

Systemic vascular resistance (SVR) = (MAP-RAP)/CO

*Vasoactive-inotropic score (VIS) is calculated using vasoactive drug doses as follows:*

VIS = dobutamine + dopamine + 10*phenylephrine + 10*milrinone + 100*epinephrine + 100*norepinephrine + 10,000*vasopressin

(Doses for all agents are in µg/kg/min with the exception of vasopressin which is in units/kg/min)

**Supplemental Table S1. Sensitivity analysis of distribution of cardiogenic shock type pooling isolated cardiogenic shock and mixed cardiogenic shock.**

| **Cardiogenic Shock Etiology** | **Primary Analysis** | **Sensitivity Analysis** | |
| --- | --- | --- | --- |
|  | **Isolated CS**  **(N=5869)** | **Isolated CS**  **(N=2318*)** | **Isolated CS + Mixed CS**  **(N=2826*)** |
| **AMI-CS, n (%)** | **27.2%** | **27.6%** | **26.5%** |
| STEMI-CS, n (%) | 65.1% | 66.7% | 65.1% |
| NSTEMI-CS, n (%) | 34.0% | 32.6% | 34.1% |
| **HF-CS, n (%)** | **59.2%** | **58.7%** | **59.2%** |
| De novo HF-CS, n (%) | 27.9% | 28.8% | 30.5% |
| Acute-on-chronic HF-CS, n (%) | 72.1% | 71.2% | 69.5% |
| **Secondary CS, n (%)** | **13.6%** | **13.7%** | **14.2%** |

*Restricted to annual cycles with complete SHARC classification details on mixed CS cases (2021-2023).

**Supplemental Table S2. Proportion of patients with mixed shock syndrome in each etiologic subtype of cardiogenic shock (based on sensitivity analysis cohort).**

| **Cardiogenic Shock Etiology** | **Mixed Shock Syndrome*** |
| --- | --- |
| **AMI-CS** | **14.8%** |
| STEMI-CS | 12.7% |
| NSTEMI-CS | 18.8% |
| **HF-CS** | **18.7%** |
| De novo HF-CS | 23.3% |
| Acute-on-chronic HF-CS | 16.7% |
| **Secondary CS** | **20.9%** |

*Restricted to annual cycles with complete SHARC classification details on mixed CS cases (2021-2023).

**Supplemental Figure S1.** **SCAI shock stage by shock profile and etiology.** AMI-CS = acute myocardial infarction-related cardiogenic shock; CS = cardiogenic shock; HF-CS = heart failure-related cardiogenic shock; SCAI = Society for Cardiovascular Angiography and Intervention.


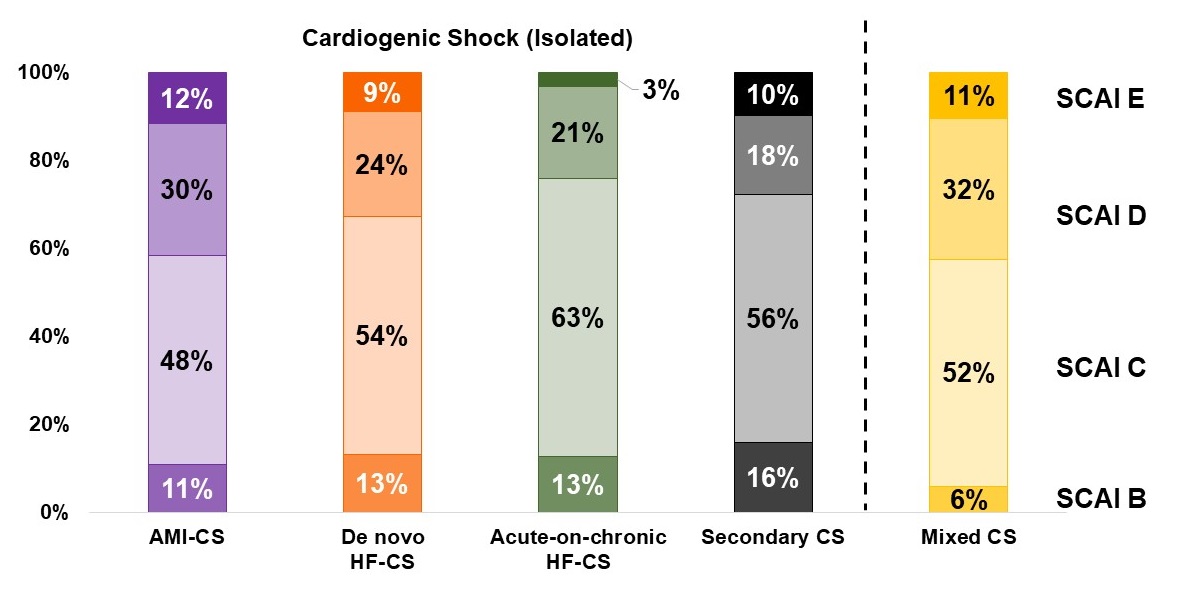


**Supplemental Figure S2**. **Sensitivity analysis of distribution of cardiogenic shock type pooling isolated cardiogenic shock and mixed shock with both cardiogenic and distributive elements.** AMI-CS = acute myocardial infarction-related cardiogenic shock; CS = cardiogenic shock; HF-CS = heart failure-related cardiogenic shock.


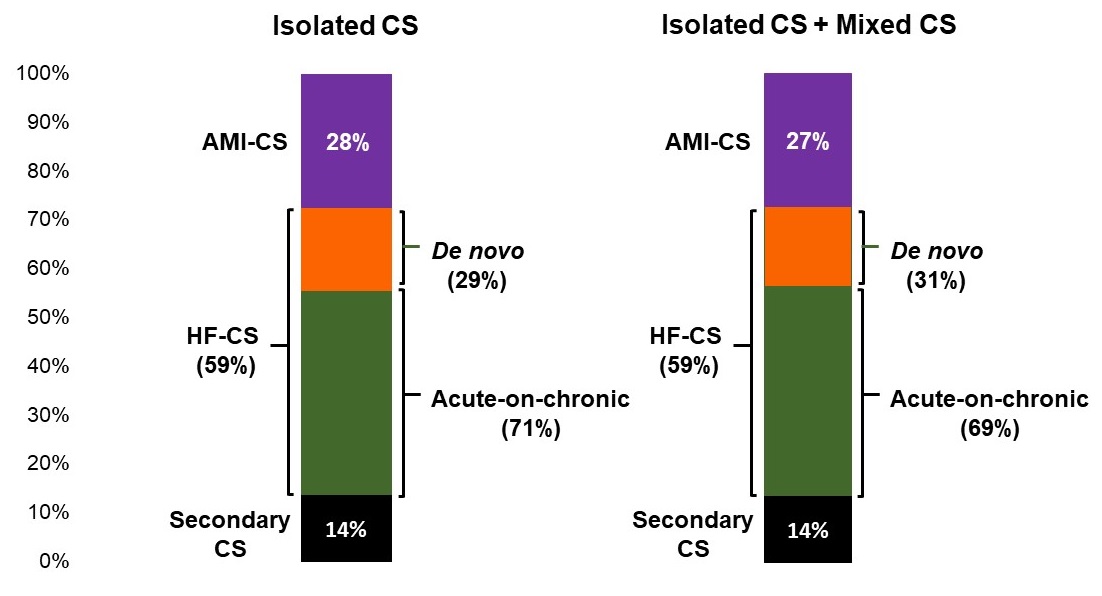


**Supplemental Figure S3.** **In-hospital mortality by SCAI stage across cardiogenic shock categories.** AMI-CS = acute myocardial infarction-related cardiogenic shock; CS = cardiogenic shock; HF-CS = heart failure-related cardiogenic shock.

**
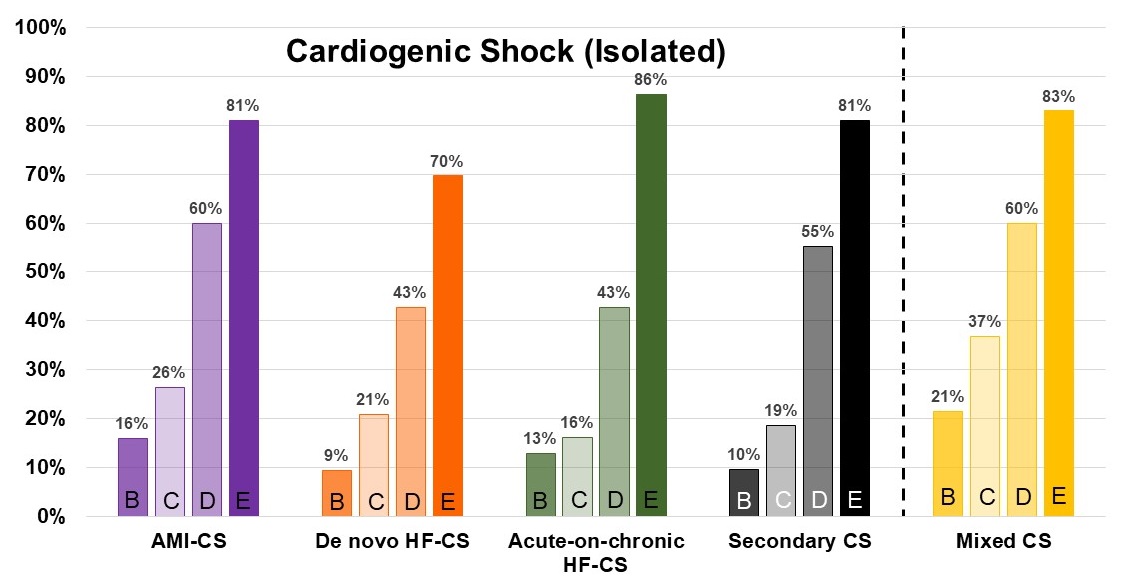
**
